# Impact of Ultraviolet-Based Combined Disinfection Processes on the Formation and Toxicity of Ciprofloxacin Disinfection Byproducts in Water

**DOI:** 10.3390/toxics13110995

**Published:** 2025-11-19

**Authors:** Yang Guo, Chengyu Zhou, Tao Zhu, Kangle Shao, Junhao Wang

**Affiliations:** School of Environmental Science and Engineering, Changzhou University, Changzhou 213614, China

**Keywords:** ultraviolet-based combined disinfection, ciprofloxacin, disinfection byproducts, *Microcystis aeruginosa*, toxicity

## Abstract

Fluoroquinolones (FQs) are ubiquitously present in aquatic environments, garnering considerable research attention. Ciprofloxacin (CIP), the most extensively utilized FQ antibiotic, features high aqueous residual levels and ranks among the most frequently detected antibiotics in environmental matrices. It also acts as a precursor of disinfection byproducts (DBPs). In recent years, ultraviolet-based combined disinfection has been widely used. This study investigated the removal efficiency of CIP and the identification of DBPs under four disinfection systems: UV irradiation, UV/PS, UV/CaO_2_, and UV/H_2_O_2_. *Microcystis aeruginosa* (*M. aeruginosa*), a dominant algal species in eutrophic freshwater ecosystems, was selected as the test organism to investigate the toxicity of DBPs generated via distinct disinfection approaches. The results demonstrated significant variations in CIP removal efficiency among the four disinfection methods. The removal rates reached 93–99% under UV/H_2_O_2_, UV/CaO_2_, and UV/PS, while single UV irradiation achieved only 87%. Sixteen DBPs were identified during the process. The DBPs produced under different disinfection methods exhibited varying inhibitory effects on *M. aeruginosa* growth. DBPs formed under the UV/H_2_O_2_ and UV/CaO_2_ systems displayed the strongest inhibition, with maximum inhibition rates of 42.1% and 36.2% within 12 days, respectively. In contrast, DBPs formed under the UV/PS and UV systems showed weaker inhibition (25.3% and 22.1%, respectively), and their inhibitory effects decreased at higher disinfection byproduct (DBP) concentrations. The results indicate that while combined UV disinfection enhances CIP removal, it may also increase the toxicity of the resulting DBPs to aquatic organisms. Overall, the UV/PS process demonstrated the highest degradation efficiency for CIP and produced disinfection byproducts (DBPs) with lower toxicity, making it the most effective and environmentally friendly method for treating water contaminated with ciprofloxacin.

## 1. Introduction

Disinfection is an essential process in water treatment. Among various methods, UV-based combined disinfection has seen increasingly widespread application in recent years. These advanced oxidation processes (AOPs) rely on the generation of highly reactive radicals such as ·OH, ·SO_4_^−^, and ·O_2_^−^, which exhibit excellent pollutant removal capabilities and promising application prospects [1,2]. However, during disinfection, disinfectants may react with natural organic matter (NOM) or anthropogenic pollutants in water to form numerous disinfection byproducts (DBPs), including trihalomethanes (THMs), haloacetic acids (HAAs), haloacetonitriles, and halonitromethanes [3]. DBPs can disrupt ecosystem balance, pose threats to the aquatic environment, and endanger human health through ingestion and dermal contact. Many DBPs have been proven to be cytotoxic and genotoxic, and some exhibit carcinogenic, mutagenic, and teratogenic properties [4,5].

Antibiotics are widely present in aquatic environments [2,6]. Among them, fluoroquinolone (FQs) are a class of broad-spectrum antibiotics commonly used in clinical and agricultural settings [7]. Ciprofloxacin (CIP), a typical synthetic FQ antibiotic, exhibits high antibacterial activity, wide antimicrobial spectrum, and high mobility [8,9].

CIP concentrations can reach μg/L levels in wastewater treatment plants [10]. Due to its chemical stability and low biodegradability, CIP tends to bioaccumulate and exerts potential toxic effects on human health and ecosystems [11,12,13]. Conventional water treatment processes cannot completely remove CIP [13,14]. Traditional activated sludge methods show removal rates of 56–75% for quinolones, with CIP having the lowest removal efficiency of only 27–44% [15,16]. Therefore, CIP serves as an important precursor compound, with the potential to generate numerous DBPs during disinfection (such as THMs and HAAs [17,18]). It is worth noting that in recent years, AOPs have received significant attention for the treatment of antibiotic wastewater [19,20]. Studies have reported that when the CIP dosage is higher, the ability of microorganisms to degrade intermediate products decreases, suggesting that more toxic intermediate products may be formed [21,22].

Algae, as primary producers in aquatic ecosystems, play a crucial role in ecological balance. *M. aeruginosa* is a representative cyanobacteria whose growth status affects higher trophic levels and overall ecosystem stability. Its ecological relevance and ease of cultivation make it a suitable model organism for aquatic toxicity studies [23]. As it is a dominant species in freshwater eutrophic waters with a key ecological role, the experimental results are ecologically representative. Moreover, it is sensitive to DBPs, with a clear concentration–effect relationship, allowing for accurate assessment of the toxicity of DBPs and the safety of the process during CIP degradation [24,25]. Once DBPs are discharged into natural water bodies, they may exert toxic effects on algae. Previous studies have mainly focused on the toxicity of antibiotics themselves, with limited research on the toxicity of antibiotic-derived DBPs. Therefore, investigating the toxicological effects of antibiotics and their DBPs on algal growth is essential.

In this study, CIP was selected as the target contaminant to evaluate its degradation behavior and DBPs formation under four disinfection systems: UV, UV/PS, UV/H_2_O_2_, and UV/CaO_2_. *M. aeruginosa* was used as a model organism to assess the toxicity. The objective of this study was to compare CIP removal efficiencies, identify DBPs transformation pathways, and assess their toxicological effects on *M. aeruginosa*.

## 2. Materials and Methods

### 2.1. Materials

All chemicals used in this study, unless otherwise noted, were purchased as reagent-grade chemicals from Shanghai Aladdin Biochemical Technology Co., Ltd. (Shanghai, China). CIP (analytical reagent-grade, AR) and CaO_2_ were purchased from Sangon Biotech Co., Ltd. (Shanghai, China). Formic acid (HPLC-grade) was also purchased from Aladdin, while methanol (HPLC-grade) was obtained from ACS Chemical Company (Columbus, OH, USA). Coomassie Brilliant Blue was purchased from Macklin Biochemical Technology Co., Ltd. (Shanghai, China). Potassium persulfate (PS, AR) and anhydrous ethanol (AR) were supplied by Sinopharm Chemical Reagent Co., Ltd. (Shanghai, China), and 30% H_2_O_2_ (AR) was purchased from Shanghai Lingfeng Chemical Reagent Co., Ltd. (Shanghai, China). All water used in the experiments was deionized water.

### 2.2. Experimental Procedures

#### 2.2.1. Degradation Kinetics of CIP and Analysis of DBP Formation Under Four Disinfection Systems

The UV disinfection experiments were conducted using a photochemical reactor system, as shown in Figure S1 of the Supplementary Material. The reactor was equipped with a 245 nm mercury lamp as the UV light source. Each reaction mixture had a total volume of 100 mL, with an initial CIP concentration of 20 μmol/L. A 0.01 M phosphate buffer solution was added to stabilize the pH of the reaction system. The reaction time was 40 min. No additional oxidant was added to the UV system. In the UV/PS, UV/H_2_O_2_, and UV/CaO_2_ systems, the corresponding oxidants (K_2_S_2_O_8_, H_2_O_2_, and CaO_2_, respectively) were added, with the initial molar ratio [CIP]_0_:[oxidant]_0_ set at 1:1, 1:3, and 1:5 to investigate the optimal ratio for maximizing CIP removal efficiency. UV intensities were set at 50 W, 100 W, and 200 W to explore the effect of light intensity on disinfection. The optimal conditions (100 W UV intensity, 1:3 molar ratio of CIP to oxidant) were used to further evaluate the influence of different initial CIP concentrations (10, 20, and 30 μmol/L) on degradation efficiency. Experiments were conducted in triplicate to ensure reliability.

The reaction solution was initially mixed using a magnetic stirrer in the dark before UV exposure. Samples of 5 mL were withdrawn at fixed intervals during the 40 min reaction and quenched immediately with 70 μL of methanol [26,27]. Then, the samples were filtered through a 0.22 μm aqueous filter membrane.

#### 2.2.2. Toxicity of CIP and Its DBPs on *M. aeruginosa*

*M. aeruginosa* (strain FACHB-905) was obtained from the Freshwater Algae Culture Collection at the Institute of Hydrobiology, Chinese Academy of Sciences. The algal cells were pre-cultured in BG11 medium under sterile conditions in 250 mL flasks. The BG11 medium was sterilized at 121 °C for 15 min in an autoclave and cooled to room temperature before use. The algae were cultured at 25 ± 1 °C under a light intensity of 3000 Lux, with alternating 12 h light and dark cycles. The flasks were shaken three times daily to prevent algal cell sedimentation. Toxicity tests commenced when the algal cells reached the logarithmic growth phase.

Under aseptic conditions, pre-cultured algal suspensions were inoculated into 250 mL flasks containing BG-11 medium to an initial optical density (OD_680_) of 0.1. Then, 0.1, 0.5, and 1 mL of the mixed reaction solution containing CIP and its DBPs (treated for 40 min by single UV, UV/PS, UV/H_2_O_2_, and UV/CaO_2_ disinfection methods, with an initial CIP concentration of 20 μmol/L and UV intensity of 100 W) were added to the respective flasks. Each treatment was performed in triplicate. The culture conditions are shown in Table S1 of the Supplementary Material.

### 2.3. Analytical Methods

#### 2.3.1. Determination of CIP Concentration

CIP concentrations were measured using a high-performance liquid chromatography (HPLC) system (Agilent 1260, Santa Clara, CA, USA) equipped with an Agilent Zorbax SB-C18 column (2.1 mm × 100 mm, 1.8 μm) [27,28]. Mobile phases A and B were 0.1% formic acid in water and methanol (formic acid was purchased from Shanghai Aladdin Biochemical Technology Co., Ltd., and methanol was purchased from Anachem Chemical Supply Company (Columbus, OH, USA, AR-grade), respectively, with a flow rate of 0.3 mL/min and a column temperature of 30 °C. The injection volume was 5 μL. The measurement time for each sample was set to 7 min. The gradient program for mobile phases A and B was 0–3 min, 80:20 (A:B); 3–5 min, 70:30 (A:B); 5–7 min, and 80:20 (A:B). The CIP peak appeared at 5.5 min.

#### 2.3.2. Determination of DBPs

DBPs were determined using a Thermo Scientific Q-Exactive high-resolution liquid chromatography mass spectrometer (LC-MS/MS), equipped with a Shimadzu InertSustain-C18 column (2.1 × 50 mm, 1.9 μm). Mobile phases A and B were 0.1% formic acid in water and 0.1% formic acid in methanol, respectively, with a flow rate of 0.2 mL/min. The elution program was 0–2 min, 90% A; 2–6.5 min, 90% A to 10% A; 6.5–10 min, 10% A; 10.0–10.1 min, 10% A to 90% A; 10.1–14.0 min, 90% A. Mass spectrometry conditions were as follows: ion transfer tube temperature 350 °C, vaporizer temperature 300 °C, sheath gas pressure 35 arb, auxiliary gas pressure 10 arb, and spray voltage 3.5 kV. Data were collected in both positive and negative ionization modes using Full MS-ddMS^2^ within an m/z range of 50–750, with an HCD collision energy of 25 eV for the top five ions.

#### 2.3.3. Measurement of Algal Growth and Physiological/Biochemical Indicators

During the 12-day cultivation period, algal growth was monitored daily by measuring the optical density (OD_680_) using a UV–Vis spectrophotometer to evaluate cell density changes. On days 6 and 12, chlorophyll a and enzyme activities were determined. For chlorophyll a, 4 mL of algal suspension was centrifuged at 8000 rpm for 10 min at 25 °C to collect the pellet, which was then resuspended in 4 mL of methanol and stored at 4 °C for 24 h. The extract was centrifuged again at 8000 rpm for 10 min, and the supernatant was analyzed for absorbance at 652 nm and 665 nm. Chlorophyll a concentration (C_a_, mg/L) was calculated according to Equation (1):

C_a_ = 16.82 × A_665_ − 9.28 × A_652_, (1)

For enzyme assays, 30 mL of algal suspension was centrifuged at 4000 rpm for 15 min, and the pellet was resuspended in 20 mL of pre-chilled (4 °C) 0.01 mol/L phosphate buffer (pH 7.4). The suspension was ultrasonically disrupted in an ice bath for 3 min (900 W, 5 s pulse, 5 s interval). The disrupted cell mixture was centrifuged at 4000 rpm for 15 min at room temperature, and the supernatant (crude enzyme extract) was collected. It was used immediately to determine protein concentration, superoxide dismutase (SOD) activity, catalase (CAT) activity, and malondialdehyde (MDA) content. All biochemical indicators were measured using commercial assay kits (Nanjing Jiancheng Bioengineering Institute, Nanjing, China) according to the manufacturer’s instructions.

## 3. Results and Discussion

### 3.1. Effects of Different Disinfection Processes on CIP Degradation

#### 3.1.1. Degradation of CIP Under Single UV Irradiation

**Figure 1:**
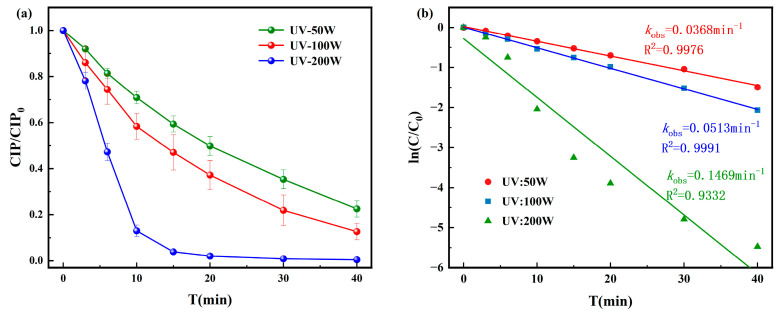
Degradation of CIP by (**a**) UV and corresponding pseudo-first-order degradation kinetics for (**b**) UV.

The degradation results of CIP under UV disinfection are shown in Figure 1. The degradation process of CIP followed a pseudo-first-order kinetic model. When the UV intensity was 200 W, CIP was almost completely degraded within 40 min, with a pseudo-first-order rate constant of 0.1469 min^−1^. Under 50 W and 100 W, the removal efficiencies after 40 min were 75% and 88%, with corresponding pseudo-first-order rate constants of 0.0368 min^−1^ and 0.0513 min^−1^, respectively. These results indicate that CIP degradation efficiency increases with UV intensity, but complete removal cannot be achieved under UV irradiation alone. The reason is that UV alone, without auxiliary oxidation, cannot generate strong radicals like ·OH and can only break weak bonds, making it difficult to destroy the stable parent nucleus and likely leaving toxic intermediates. Therefore, CIP cannot be completely removed.

#### 3.1.2. Degradation of CIP Under the UV/H_2_O_2_ System

**Figure 2:**
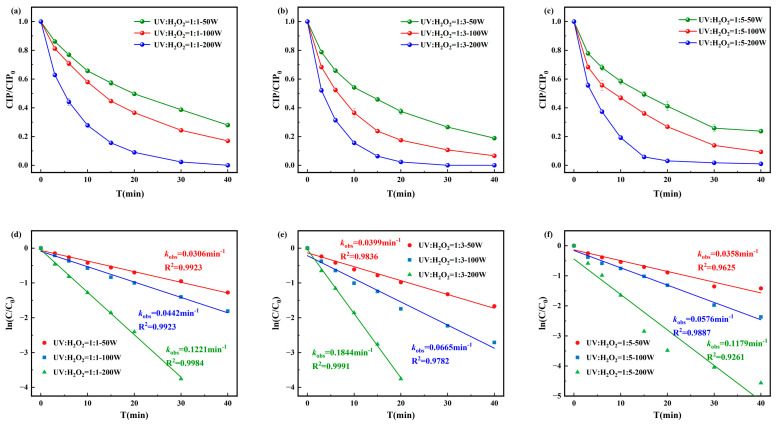
Degradation of CIP by (**a**) [CIP]_0_:[H_2_O_2_]_0_ = 1:1, (**b**) [CIP]_0_:[H_2_O_2_]_0_ = 1:3, and (**c**) [CIP]_0_:[H_2_O_2_]_0_ = 1:5 and corresponding pseudo-first-order degradation kinetics for (**d**) [CIP]_0_:[H_2_O_2_]_0_ = 1:1, (**e**) [CIP]_0_:[ H_2_O_2_]_0_ = 1:3, and (**f**) [CIP]_0_:[H_2_O_2_]_0_ = 1:5.

The degradation efficiency and pseudo-first-order kinetics of CIP under the UV/H_2_O_2_ process are presented in Figure 2. The highest degradation efficiency was achieved when the molar ratio of [CIP]_0_:[H_2_O_2_]_0_ was 1:3 and the UV intensity was 200 W, with complete degradation occurring within 30 min and a pseudo-first-order rate constant of 0.1844 min^−1^. When [CIP]_0_:[H_2_O_2_]_0_ = 1:1, the pseudo-first-order rate constant ranged from 0.0306 to 0.1221 min^−1^, while for 1:5, it ranged from 0.0358 to 0.1179 min^−1^. These results suggested that excess H_2_O_2_ can act as a quencher of ·OH, consuming the highly reactive ·OH in the system through reactions and producing ·OH with weaker oxidative ability. This not only reduces the number of ·OH attacking CIP and its intermediates but also decreases the overall oxidation efficiency, ultimately leading to a decline or even inhibition of the CIP degradation rate.

#### 3.1.3. Degradation of CIP Under the UV/CaO_2_ System

**Figure 3:**
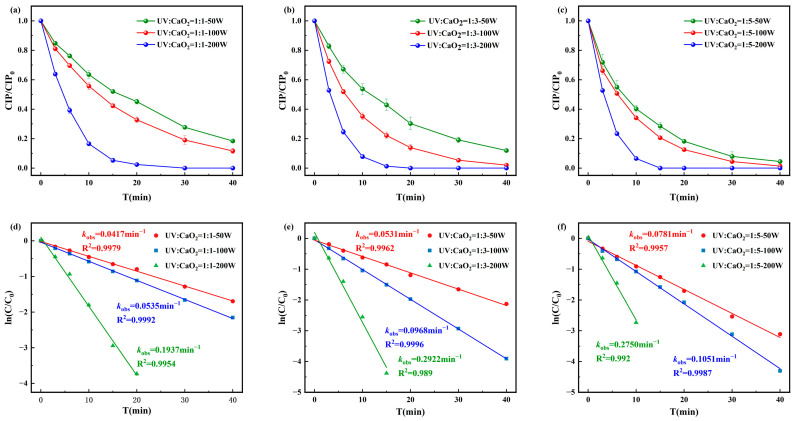
Degradation of CIP by (**a**) [CIP]_0_:[CaO_2_]_0_ = 1:1, (**b**) [CIP]_0_:[CaO_2_]_0_ = 1:3, and (**c**) [CIP]_0_:[CaO_2_]_0_ = 1:5 and corresponding pseudo-first-order degradation kinetics for (**d**) [CIP]_0_:[CaO_2_]_0_ = 1:1, (**e**) [CIP]_0_:[CaO2]_0_ = 1:3, and (**f**) [CIP]_0_:[CaO_2_]_0_ = 1:5.

As shown in Figure 3, the degradation efficiency of CIP under the UV/CaO_2_ system increased with both UV intensity and CaO_2_ concentration. When [CIP]_0_:[CaO_2_]_0_ = 1:5 and UV intensity = 200 W, CIP was completely degraded within 15 min, with a maximum pseudo-first-order rate constant is 0.275 min^−1^ and R^2^ = 0.992. At lower oxidant ratios (1:1 and 1:3), the pseudo-first-order rate constants ranged from 0.0417 to 0.1937 min^−1^ and from 0.0531 to 0.2922 min^−1^, respectively. Based on the comprehensive experimental results, [CIP]_0_:[CaO_2_]_0_ = 1:3 and UV intensity = 100 W were selected as optimal conditions for subsequent experiments.

#### 3.1.4. Degradation of CIP Under the UV/PS System

**Figure 4:**
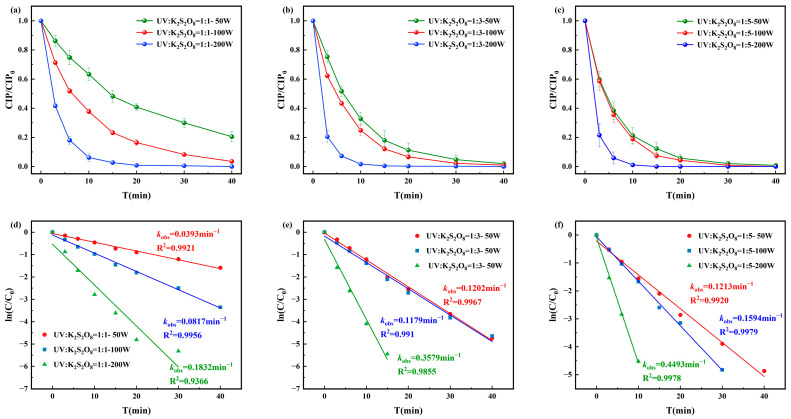
Degradation of CIP by (**a**) [CIP]_0_:[PS]_0_ = 1:1, (**b**) [CIP]_0_:[PS]_0_ = 1:3, and (**c**) [CIP]_0_:[PS]_0_ = 1:5 and corresponding pseudo-first-order degradation kinetics for (**d**) [CIP]_0_:[PS]_0_ = 1:1, (**e**) [CIP]_0_:[PS]_0_ = 1:3, and (**f**) [CIP]_0_:[PS]_0_ = 1:5.

The degradation rates of CIP by the UV/PS disinfection process and the corresponding pseudo-first-order degradation kinetics are shown in Figure 4. The degradation efficiency of CIP was positively correlated with PS dosage and UV light intensity. When [CIP]_0_:[PS]_0_ = 1:1, the pseudo-first-order rate constant ranged from 0.0393–0.1832 min^−1^; at 1:3, it increased to 0.1202–0.3579 min^−1^. Under [CIP]_0_:[PS]_0_ = 1:5 and UV = 200 W, complete degradation was achieved within 15 min with a pseudo-first-order rate constant of 0.4493 min^−1^. The most stable and efficient performance was observed at [CIP]_0_:[PS]_0_ = 1:3 and UV = 100 W, which was therefore selected as the optimal condition for subsequent kinetic and toxicity analyses.

#### 3.1.5. Degradation of CIP at Different Initial Concentrations

Under the optimal conditions (UV = 100 W, [CIP]_0_:[oxidant]_0_ = 1:3), the pseudo–first-order degradation kinetics of CIP at initial concentrations of 10, 20, and 30 μmol/L were examined for the three combined systems (UV/H_2_O_2_, UV/CaO_2_, and UV/PS), as shown in Figure S2. The pseudo-first-order rate constants gradually decreased with increasing initial CIP concentration. The maximum pseudo-first-order rates under UV/H_2_O_2_, UV/CaO_2_, and UV/PS disinfection were 0.0749 min^−1^, 0.0998 min^−1^, and 0.1279 min^−1^, respectively. These results indicated that the degradation efficiency followed the order UV/PS > UV/CaO_2_ > UV/H_2_O_2_, confirming that UV/PS provided the most efficient photochemical degradation pathway for CIP.

### 3.2. Formation of DBPs During CIP Degradation

A total of 16 DBPs from CIP were identified by HPLC-MS during the four disinfection processes (UV, UV/H_2_O_2_, UV/CaO_2_, and UV/PS). The generation details of DBPs are provided in Table S2 and Figure S3 of the Supplementary Material. The peak areas of the main intermediates P1 and P2 are shown in Figure S4 of the Supplementary Material. It can be seen that the UV/PS process shows the optimal inhibition effect on the formation of the main intermediates P1 and P2. Compared with the UV process alone, the inhibition rates for the formation of P1 and P2 reach 78.69% and 78.93%, respectively. The results revealed that the UV/PS process effectively suppresses DBPs formation. Considering with its superior CIP degradation efficiency, UV/PS exhibited the most balanced performance between degradation and environmental safety.

**Figure 5:**
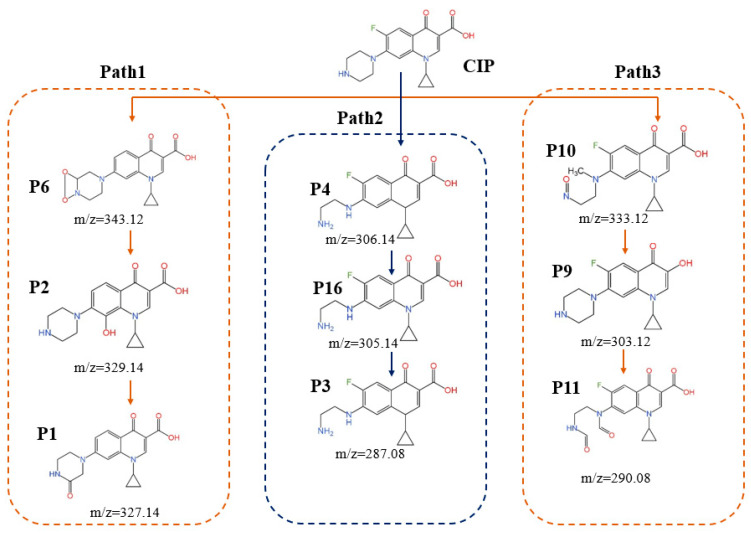
Degradation pathway of CIP in the UV/PS system.

As illustrated in Figure 5, the degradation pathways of CIP in the UV/PS process can be categorized into three major types: oxidative modification and defluorination, piperazine ring oxidation and degradation, and ring cleavage and fragmentation [29,30]. Pathway 1 centers on defluorination reactions, with free radicals preferentially attacking the quinolone ring, initially producing the intermediate P6, which then undergoes hydroxylation and carbonylation reactions to generate P2. P2 further reacts, leading to the opening and fragmentation of the quinolone ring, ultimately forming smaller molecules, P1. Pathway 2 follows the piperazine oxidation mechanism; ·SO_4_^−^ or ·OH radicals attack the piperazine ring to form the intermediate product P4. Subsequently, the piperazine ring partially breaks and gradually loses fluorine, forming P16. P16 continues to react, further simplifying the molecular structure, and ultimately generating P3. Pathway 3 involves ring cleavage and fragmentation. In this pathway, free radicals attack multiple sites, causing mild oxidation and structural modifications of the piperazine ring or quinolone ring. Initially, intermediate product P10 is formed, which then undergoes structural modifications to generate P9. P9 continues to react and eventually forms P11. Meanwhile, during the degradation process, various isomers are produced (for example, P7 and P15, P8 and P14). These isomers further degrade through cleavage of the piperazine ring or opening of the quinolone ring.

### 3.3. Effects of CIP and Its DBPs on M. aeruginosa

#### 3.3.1. Effects on Algal Growth

**Figure 6:**
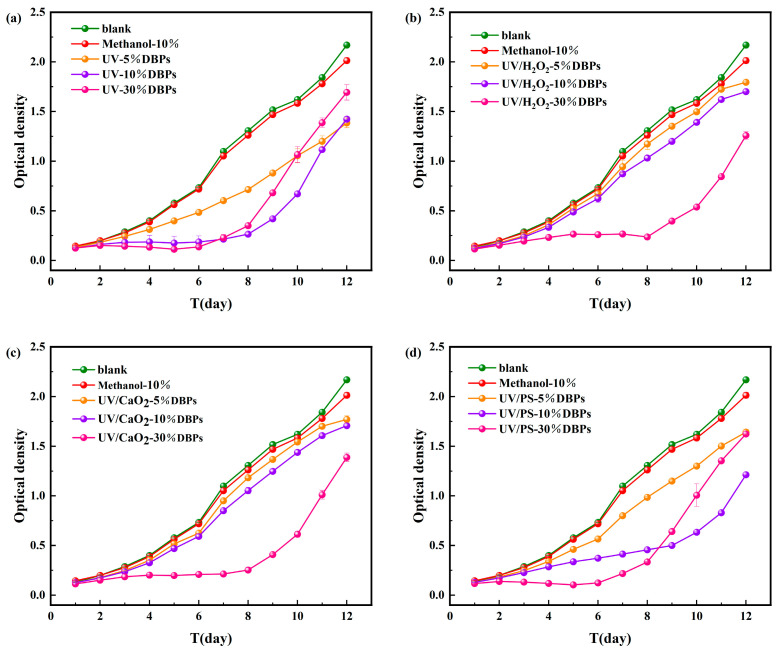
Microalgal growth inhibition after 12-day exposure to DBPs from (**a**) UV, (**b**) UV/H_2_O_2_, (**c**) UV/CaO_2_, (**d**) UV/PS.

As shown in Figure 6, during the 12-day cultivation period of *M. aeruginosa*, DBPs generated from CIP treated by the four disinfection methods all affected the growth of *M. aeruginosa* to varying degrees. Methanol (used as a quenching agent) showed negligible effects, confirming that the observed growth inhibition resulted from DBPs. Statistical significance tests for different treatments are shown in Figure S5 of the Supplementary Materials.

Under UV treatment (Figure 6a), all tested DBP concentrations (5%, 10%, 30%) inhibited algal growth over the 12-day period. During the early to mid-cultivation stage, inhibition became more pronounced with increasing concentration. On day 6, the cell dry weight of *M. aeruginosa* in the 5%, 10%, and 30% concentration groups decreased by 33%, 74%, and 81%, respectively, indicating concentration-dependent inhibition. During the later growth phase, the inhibitory effect was significantly alleviated. The cell dry weight in the 30% concentration group was only 22% lower than the control. This might be due to activation of detoxification mechanisms or DBPs transformation by the algae. Similar trends were observed under the UV/PS system (Figure 6d). In contrast, under UV/H_2_O_2_ and UV/CaO_2_ systems (Figure 6b,c), inhibition increased with DBP concentration throughout the 12-day period. The highest inhibition rates (36.05% and 42.3%, respectively) were observed on day 6 for the 30% concentration groups. The persistent inhibition under these two systems might result from the generation of DBPs with higher toxicity or slower degradation rates.

#### 3.3.2. Effects of CIP and DBPs on the Physiological and Biochemical Parameters of *M. aeruginosa*

**Figure 7:**
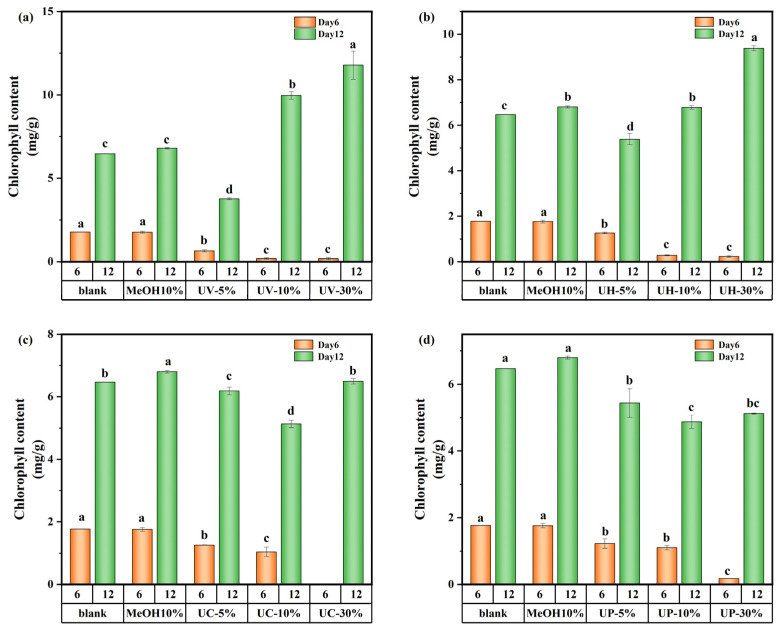
Chlorophyll a content of *M. aeruginosa* exposed to DBPs from (**a**) UV, (**b**) UV/H_2_O_2_, (**c**) UV/CaO_2_, and (**d**) UV/PS (*p* < 0.05). In (**a**–**d**), different lowercase letters (a, b, c, d) indicate statistically significant differences between groups (*p* < 0.05).

The variations in chlorophyll a of *M. aeruginosa* are shown in Figure 7. On day 6, the effect of DBPs generated by the four disinfection methods on chlorophyll a was consistent with their growth inhibition patterns, showing a decreasing trend with increasing concentration of CIP and its DBPs. At a 5% DBP concentration, the concentration of chlorophyll a was 29.1–63.5% lower than the control. This might be due to the short-term stress state of algal cells exposed to DBPs in the early cultivation stage, where the damaging effect of toxic substances on the photosynthetic system far exceeded the cells’ self-repair capacity. Higher DBP concentrations intensify this inhibition. However, by day 12, under UV and UV/H_2_O_2_ treatments, the concentration of chlorophyll a at high DBP concentrations surpassed that of the control by 82.3% and 45.2%, respectively. This might be due to long-term adaptive responses, where *M. aeruginosa* adjusts its metabolic and photosynthetic processes to tolerate or utilize the DBPs after prolonged exposure.

**Figure 8:**
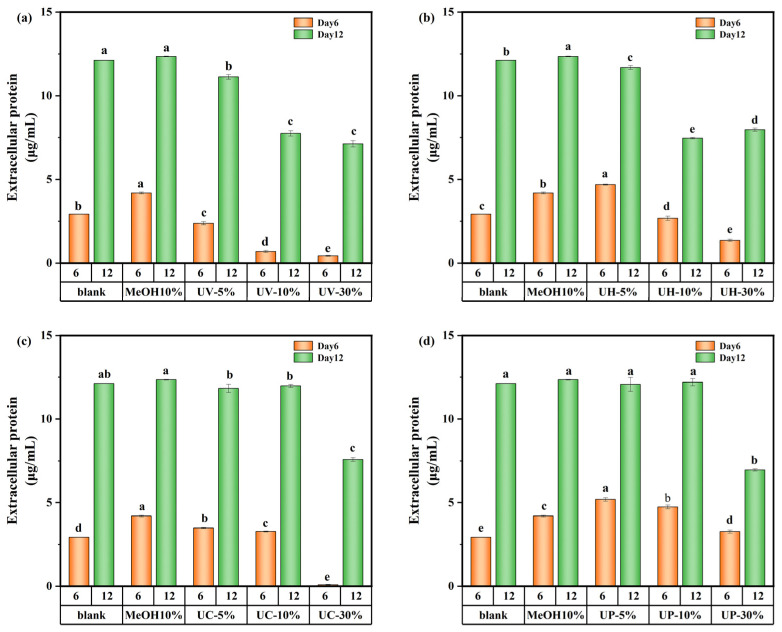
Extracellular protein content of *M. aeruginosa* exposed to DBPs from (**a**) UV, (**b**) UV/H_2_O_2_, (**c**) UV/CaO_2_, and (**d**) UV/PS (*p* < 0.05). In (**a**–**d**), different lowercase letters (a, b, c, d, e) indicate statistically significant differences between groups (*p* < 0.05).

The variations in extracellular protein content on days 6 and 12 of cultivation are shown in Figure 8. On day 6, the extracellular protein content in the UV system decreased by 18.8% at a 5% DBP concentration and showed a decreasing trend with increasing DBP concentration. In contrast, under the UV/PS, UV/H_2_O_2_, and UV/CaO_2_ disinfection systems, the extracellular protein content at low DBP concentrations was 77.2%, 60.3%, and 19%, higher than the control, respectively. However, with increasing concentration, the extracellular protein content decreased significantly. These results suggest that during the early exposure stage, high DBP concentrations directly inhibited intracellular protein synthesis pathways and reduced extracellular protein secretion.

By day 12, under the UV/CaO_2_ and UV/PS disinfection methods, the extracellular protein content showed a different appearance. The medium-concentration group (10%) had the highest extracellular protein content compared to the low-concentration (5%) and high-concentration (30%) groups. This was because the medium-concentration group experienced moderate stress from DBPs in the early stage, potentially stimulating some latent synthesis mechanisms within the cells, leading to the highest extracellular protein content. Overall, although transient stimulatory effects occurred at specific concentrations and culture time, DBPs generated under all disinfection systems (UV, UV/PS, UV/H_2_O_2_, UV/CaO_2_) demonstrated an inhibitory impact on protein synthesis over the 12-day cultivation period.

**Figure 9:**
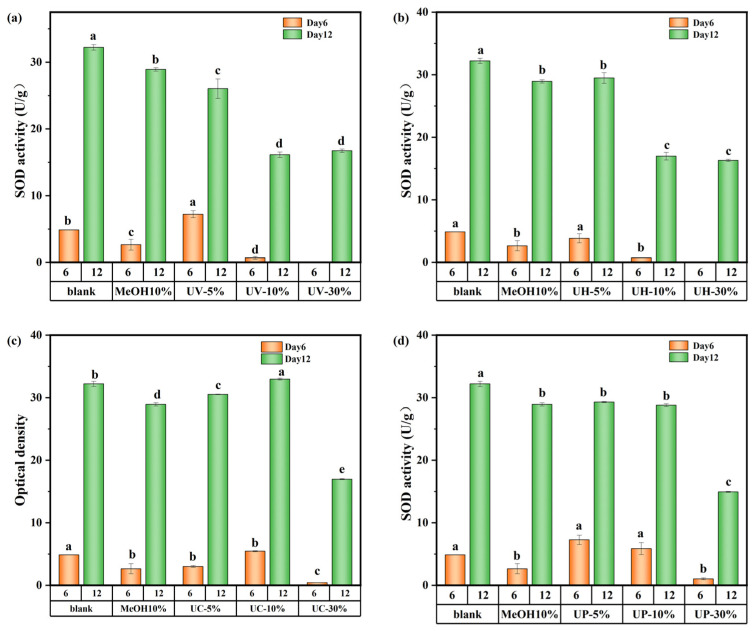
SOD activity value of *M. aeruginosa* exposed to DBPs from (**a**) UV, (**b**) UV/H_2_O_2_, (**c**) UV/CaO_2_, and (**d**) UV/PS (*p* < 0.05). In (**a**–**d**), different lowercase letters (a, b, c, d, e) indicate statistically significant differences between groups (*p* < 0.05).

The effects of CIP and its DBPs on SOD and CAT activities are presented in Figure 9 and Figure S6 of the Supplementary Material, respectively. As shown in Figure 9a,b, under UV and UV/H_2_O_2_ treatments, SOD activity in the high-concentration (30%) groups initially decreased but gradually recovered by day 12, approaching levels similar to those of the medium-concentration (10%) groups but still below that of the low-concentration (5%) groups. Specifically, on days 6 and 12, SOD activity in the low-concentration UV system group decreased by 22.6% and 19.2%, respectively. In the UV/H_2_O_2_ system, the decreases were 20.99% and 8.7%, respectively. This can be explained by the stress and repair mechanisms of algal cells [31,32]. On day 6, cells in the low-concentration group experienced mild toxic stress and showed localized damage but maintained high SOD synthesis efficiency with minimal enzyme consumption. In contrast, the high-concentration group suffered from excessive toxic stress, impairing SOD synthesis mechanisms and resulting in lower enzyme activity. During the late cultivation stage, algal cells in the high-concentration group gradually restored SOD synthesis capacity. However, due to residual damage from the earlier stage and the presence of DBPs in the water, the enzyme activity could only recover to the level of the medium-concentration group.

CAT activity showed trends similar to those of SOD throughout the 12-day period (Figure S6), indicating that *M. aeruginosa* upregulated SOD and CAT activities to scavenge excess reactive oxygen species (ROS) and mitigate oxidative stress. However, enzyme recovery was constrained by the extent of initial damage, and high DBP concentrations prevented full restoration of antioxidant capacity.

**Figure 10:**
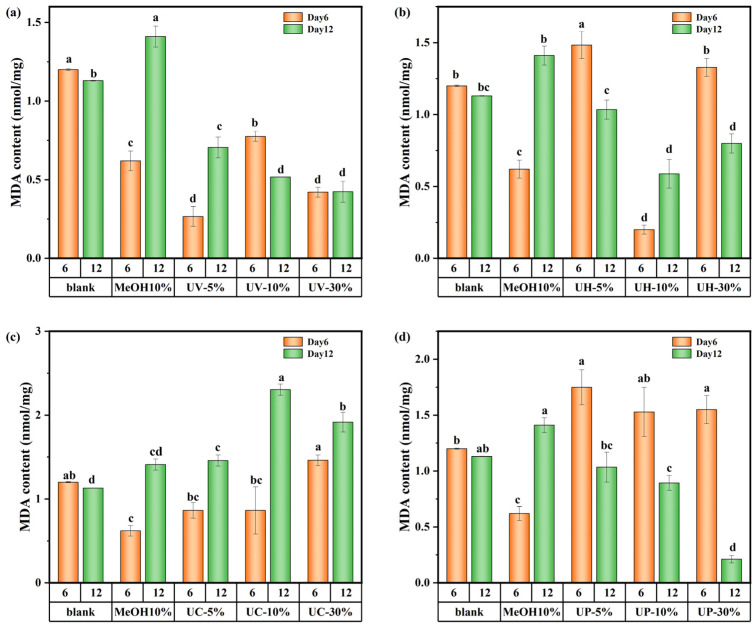
MDA content in *M. aeruginosa* exposed to DBPs from (**a**) UV, (**b**) UV/H_2_O_2_, (**c**) UV/CaO_2_, and (**d**) UV/PS (*p* < 0.05). In (**a**–**d**), different lowercase letters (a, b, c, d) indicate statistically significant differences between groups (*p* < 0.05).

The changes in the MDA content of *M. aeruginosa* are shown in Figure 10. These results indicate significant differences in the impact of CIP and its DBPs under the four disinfection systems on the MDA content of algal cells. In the UV system, the 5% DBP group showed a 96.2% increase in MDA from day 6 to day 12, while the 10% group showed a 44% decrease over the same period (Figure 10a). The trend in the UV/H_2_O_2_ system was consistent with that of the UV system (Figure 10b). It can be inferred that varying DBP concentrations lead to dominant toxic substances causing different oxidative responses across growth stages. In contrast, in the UV/CaO_2_ system (Figure 10c), all DBP concentration groups exhibited higher MDA levels on day 12 than on day 6, indicating persistent oxidative stress from highly toxic and recalcitrant DBPs that continued to damage cell membranes throughout the cultivation period. Conversely, under the UV/PS system (Figure 10d), MDA content in all concentration groups decreased over time, suggesting that the DBPs formed in the UV/PS system were of lower toxicity and more biodegradable, leading to a consequent decrease in MDA content. In summary, DBPs generated from CIP by the UV/PS disinfection process exhibited the weakest toxicity towards *M. aeruginosa.*

## 4. Conclusions

This study investigated the photochemical degradation of CIP and the formation of DBPs under four UV-based disinfection systems: UV, UV/PS, UV/CaO_2_, and UV/H_2_O_2_. The results showed that under identical experimental conditions, the photochemical degradation rates of CIP by the four disinfection methods differed significantly. The UV/PS system achieved the highest CIP degradation rate of 99%. Sixteen DBPs were identified via HPLC–MS across the four disinfection systems. Among them, the UV/PS process generated the lowest concentration of DBPs. However, DBPs produced under all systems inhibited the growth of *M. aeruginosa* to varying degrees. The UV and UV/PS systems exhibited concentration-dependent inhibition. The UV/CaO_2_ and UV/H_2_O_2_ systems showed initial inhibition followed by partial recovery during later growth stages. This is possibly due to algal adaptation or utilization of DBPs as alternative nutrient sources. The activity of SOD and CAT was enhanced under CIP and DBPs exposure. This indicates that the activation of antioxidant defense mechanisms could reduce oxidative damage.

Comprehensive analysis indicated that the UV/PS disinfection process achieved the highest CIP degradation efficiency and most effectively reduced DBP toxicity. It can be concluded that future research should explore DBP identification at environmentally relevant concentrations and evaluate chronic toxicity in multi-trophic aquatic species.

## Data Availability

The original contributions presented in this study are included in the article. Further inquiries can be directed to the corresponding author.

## Supplementary Materials

The following supporting information can be downloaded at https://www.mdpi.com/article/10.3390/toxics13110995/s1, Table S1: Cultivation conditions for *Microcystis aeruginosa*; Table S2: Intermediate products during CIP degradation were detected by LC-MS; Figure S1: Schematic diagram of the photochemical reaction system; Figure S2: Pseudo-first-order degradation kinetics of ciprofloxacin at different concentrations for (a) UV/H_2_O_2,_ (b) UV/CaO_2,_ and (c) UV/PS. Experimental conditions: The UV irradiation intensity is 100 W, and the concentration ratio of ciprofloxacin to the oxidants is 1:3; Figure S3: Fragment mass spectrometry of CIP-derived DBPs; Figure S4: Peak area chart of main intermediates P1 and P2 from (a) UV, (b) UV/H_2_O_2_, (c) UV/CaO_2_, and (d) UV/PS; Figure S5: Growth inhibition of microalgae on the 12th day of exposure to disinfection by-products, which are derived from (a) UV, (b) UV/H_2_O_2_, (c) UV/CaO_2_, and (d) UV/PS; Figure S6: CAT activity value of *Microcystis aeruginosa* exposed to DBPs from (a) UV, (b) UV/H_2_O_2_, (c) UV/CaO_2_, and (d) UV/PS.
