# Supplementary material for: Impact of Ultraviolet-Based Combined Disinfection Processes on the Formation and Toxicity of Ciprofloxacin Disinfection Byproducts in Water
Source: Toxics. 2025 Nov 19;13(11):995. doi: 10.3390/toxics13110995 (PMC12656039; doi:10.3390/toxics13110995)
Supplement: Supplementary file 1 [file toxics-13-00995-s001.zip › toxics-3989150-supplementary.pdf]

# Impact of Ultraviolet-based Combined Disinfection Processes on the Formation and Toxicity of Ciprofloxacin Disinfection Byproducts in Water

**Yang Guo [1,*], Chengyu Zhou [1], Tao Zhu [1], Kangle Shao [1] and Junhao Wang [1]**

[1] School of Environmental Science and Engineering, Changzhou University, Changzhou 213614, PR China; guoyang@cczu.edu.cn
* Correspondence:guoyang@cczu.edu.cn; Tel.: +86-188 1052 2652

**Table S1.** Cultivation conditions for Microcystis aeruginosa

| Temperature(°C) | Light intensity (Lux) | light/ dark(h) | Time(Day) |
|---|---|---|---|
| 25 ± 1 | 3000 | 12/12 | 12 |

**Table S2.**The intermediate products during the CIP degradation were detected by LC-MS

| Products number | m/z(+H) | Formula | Structures | UV | UV/$H_2O_2$ | UV/$CaO_2$ | UV/PS |
|---|---|---|---|---|---|---|---|
| P1 | 328.13 | $C_{14}H_{13}N_3O_2$ | 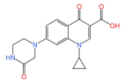 | √ | √ | √ | √ |
| P2 | 330.14 | $C_{18}H_{17}N_2O_4$ | 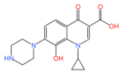 | √ | √ | √ | √ |
| P3 | 288.08 | $C_{16}H_{18}FN_3O$ | 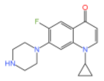 | × | √ | √ | √ |
| P4 | 307.14 | $C_{15}H_{16}FN_3O_3$ | 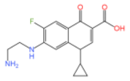 | √ | √ | √ | √ |
| P5 | 346.14 | $C_{17}H_{19}N_3O_5$ | 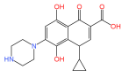 | √ | × | √ | × |
| P6 | 344.12 | $C_{17}H_{18}N_3O_5$ | 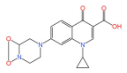 | √ | √ | √ | √ |
| P7 | 316.13 | $C_{16}H_{18}N_3O_4$ | 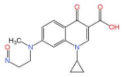 | √ | √ | √ | √ |
| P8 | 348.13 | $C_{17}H_{19}N_3O_4F$ | 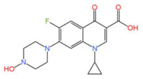 | √ | √ | √ | √ |
| P9 | 304.13 | $C_{17}H_{18}N_3O_3F$ | 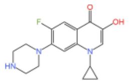 | √ | √ | √ | √ |
| P10 | 334.12 | $C_{16}H_{17}N_3O_4F$ | 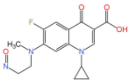 | √ | √ | √ | √ |

| | | | | | | | |
|---|---|---|---|---|---|---|---|
| P11 | 291.08 | $C_{17}H_{15}N_3O_5F$ | 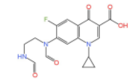 | √ | √ | √ | √ |
| P12 | 264.11 | $C_{13}H_{15}FN_3O_2$ | 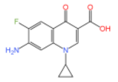 | × | × | √ | × |
| P13 | 348.13 | $C_{17}H_{19}N_3O_4F$ | 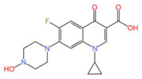 | √ | √ | √ | √ |
| P14 | 316.13 | $C_{16}H_{18}N_3O_4$ | 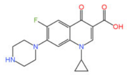 | √ | √ | √ | √ |
| P15 | 245.09 | $C_{16}H_{18}FN_3O$ | 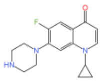 | √ | × | × | × |
| P16 | 306.12 | $C_{15}H_{17}N_3O_3F$ | 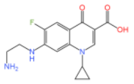 | × | √ | √ | √ |

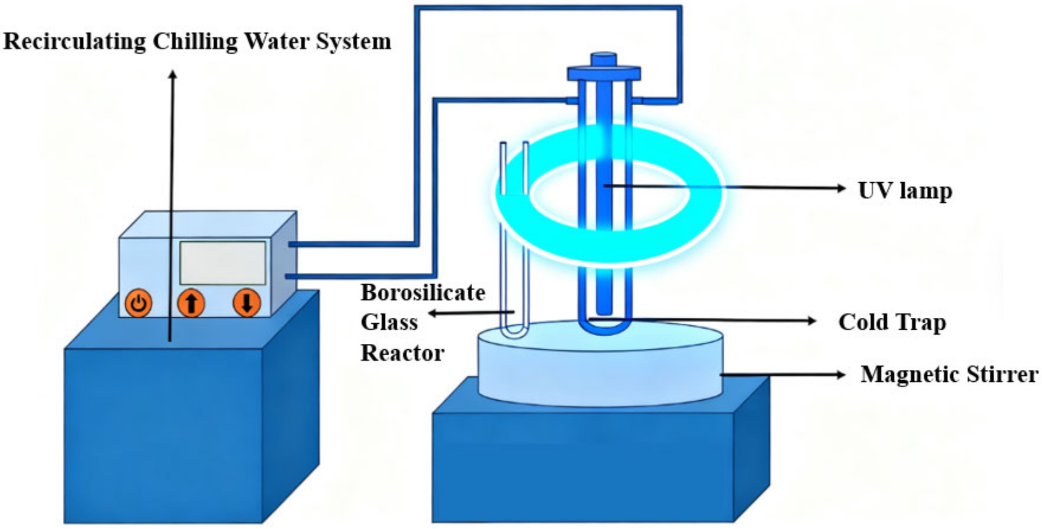


**Figure S1.** Schematic Diagram of the Photochemical Reaction System

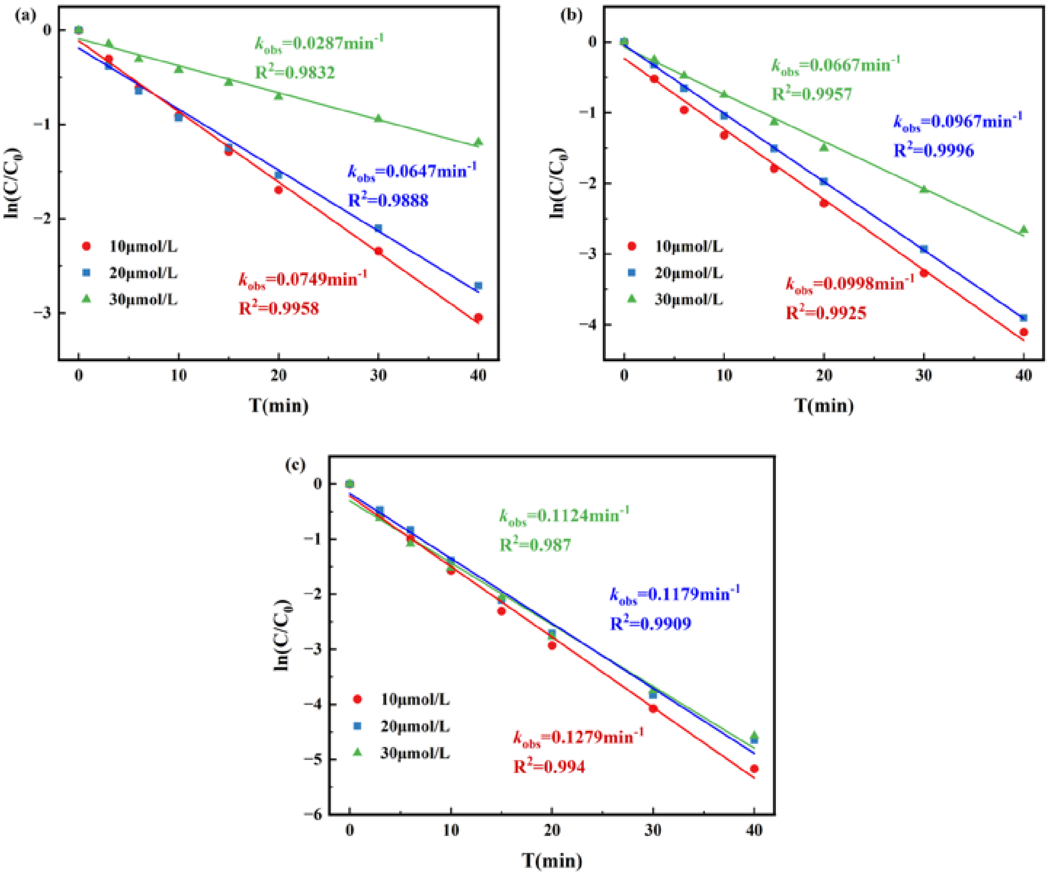


**Figure S2.** Pseudo-First-Order Degradation Kinetics of Ciprofloxacin at different concentrations for (a)UV/$H_2O_2$ (b)UV/$CaO_2$ (c)UV/PS. Experimental Conditions: The UV irradiation intensity is 100 W, and the concentration ratio of ciprofloxacin to the oxidants is 1:3.

P1

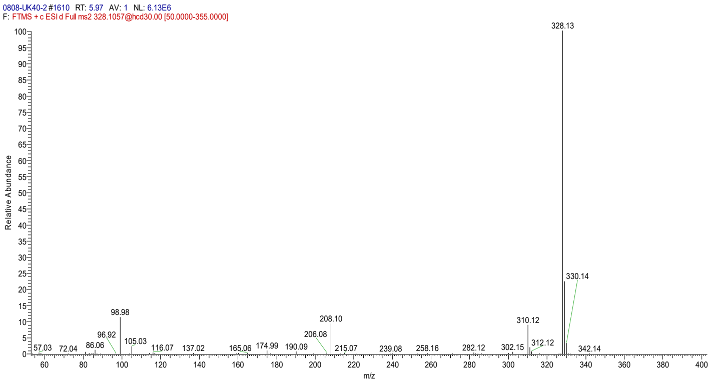


P2

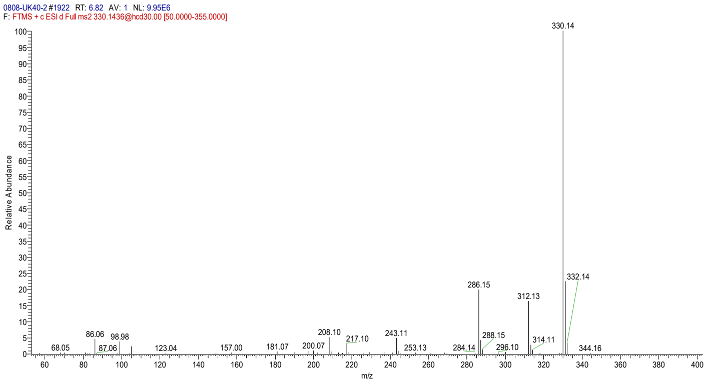
0808-UK40-2 #1922 RT: 6.82 AV: 1 NL: 9.95E6
F: FTMS + c ESI d Full ms2 330.1436@hcd30.00 [50.0000-355.0000]
Relative Abundance
m/z
330.14
332.14
286.15
312.13
208.10
217.10
243.11
288.15
314.11
86.06
98.98
68.05
87.06
123.04
157.00
181.07
200.07
253.13
284.14
296.10
344.16

P3

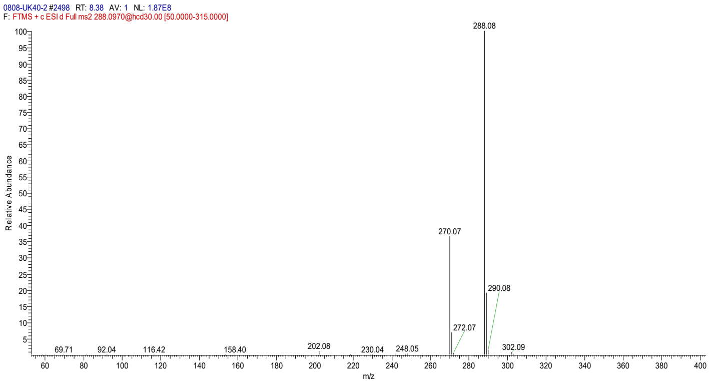
0808-UK40-2 #2498 RT: 8.38 AV: 1 NL: 1.87E8
F: FTMS + c ESI d Full ms2 288.0970@hcd30.00 [50.0000-315.0000]
Relative Abundance
m/z
288.08
270.07
290.08
272.07
69.71
92.04
116.42
158.40
202.08
230.04
248.05
302.09

P4

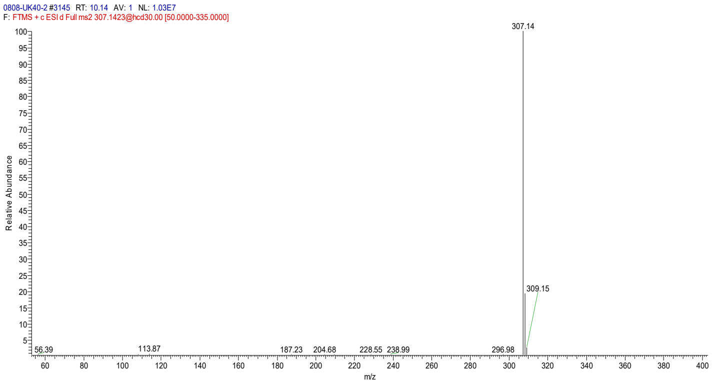
0808-UK40-2 #3145 RT: 10.14 AV: 1 NL: 1.03E7
F: FTMS + c ESI d Full ms2 307.1423@hcd30.00 [50.0000-335.0000]
Relative Abundance
m/z
307.14
309.15
56.39
113.87
187.23
204.68
228.55
238.99
296.98

P5

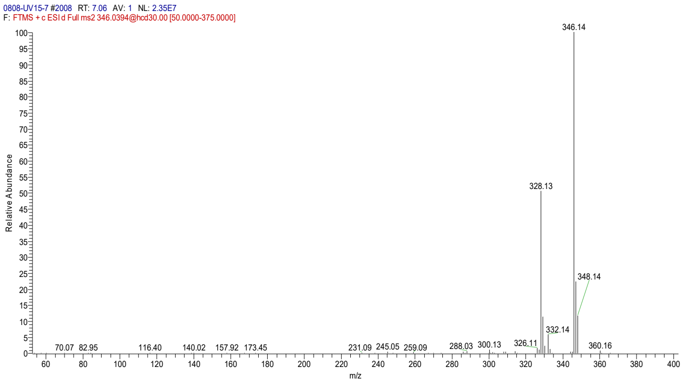
0808-UV15-7 #2008 RT: 7.06 AV: 1 NL: 2.35E7
F: FTMS + c ESI d Full ms2 346.0394@hcd30.00 [50.0000-375.0000]
Relative Abundance
m/z
346.14
328.13
348.14
332.14
70.07
82.95
116.40
140.02
157.92
173.45
231.09
245.05
259.09
288.03
300.13
326.11
360.16

P6

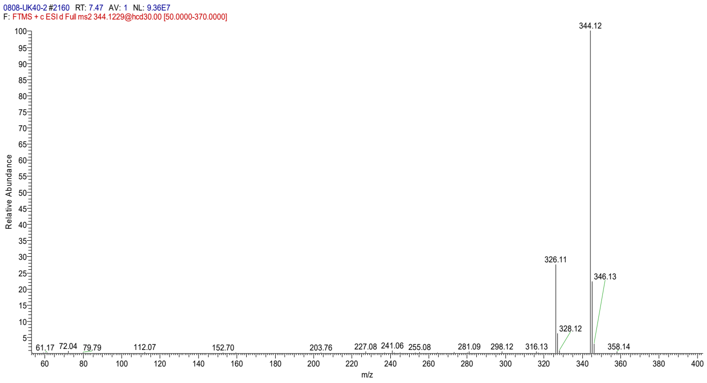
0808-UK40-2 #2160 RT: 7.47 AV: 1 NL: 9.36E7
F: FTMS + c ESI d Full ms2 344.1229@hcd30.00 [50.0000-370.0000]
Relative Abundance
m/z
344.12
326.11
346.13
328.12
61.17 72.04 79.79 112.07 152.70 203.76 227.08 241.06 255.08 281.09 298.12 316.13 358.14

P7

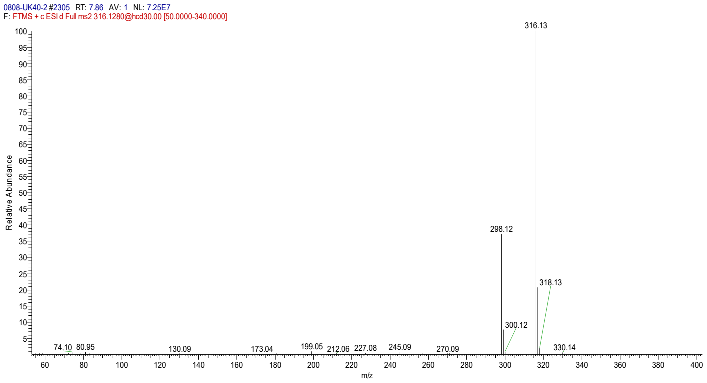
0808-UK40-2 #2305 RT: 7.86 AV: 1 NL: 7.25E7
F: FTMS + c ESI d Full ms2 316.1280@hcd30.00 [50.0000-340.0000]
Relative Abundance
m/z
316.13
298.12
318.13
300.12
74.10 80.95 130.09 173.04 199.05 212.06 227.08 245.09 270.09 330.14

P8

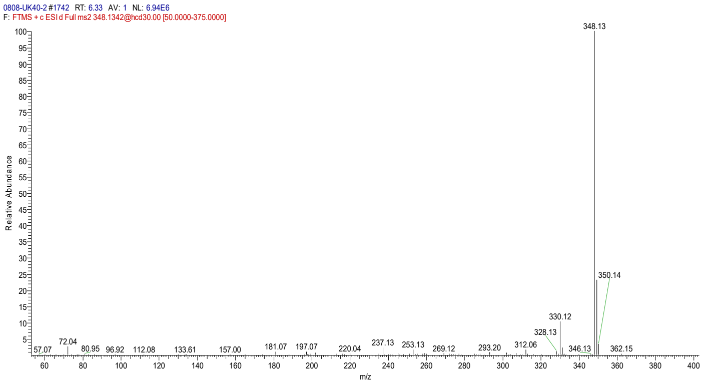
0808-UK40-2 #1742 RT: 6.33 AV: 1 NL: 6.94E6
F: FTMS + c ESI d Full ms2 348.1342@hcd30.00 [50.0000-375.0000]
Relative Abundance
m/z
348.13
350.14
330.12
328.13
57.07 72.04 80.95 96.92 112.08 133.61 157.00 181.07 197.07 220.04 237.13 253.13 269.12 293.20 312.06 346.13 362.15

P9

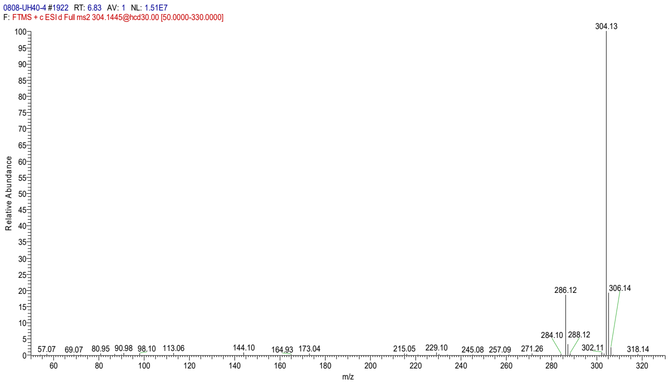
0808-UH40-4 #1922 RT: 6.83 AV: 1 NL: 1.51E7
F: FTMS + c ESI d Full ms2 304.1445@hcd30.00 [50.0000-330.0000]
Relative Abundance
m/z
304.13
286.12
306.14
284.10 288.12
57.07 69.07 80.95 90.98 98.10 113.06 144.10 164.93 173.04 215.05 229.10 245.08 257.09 271.26 302.11 318.14

P10

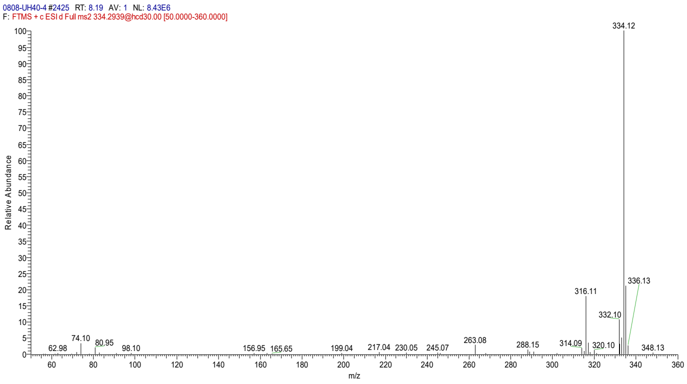


P11

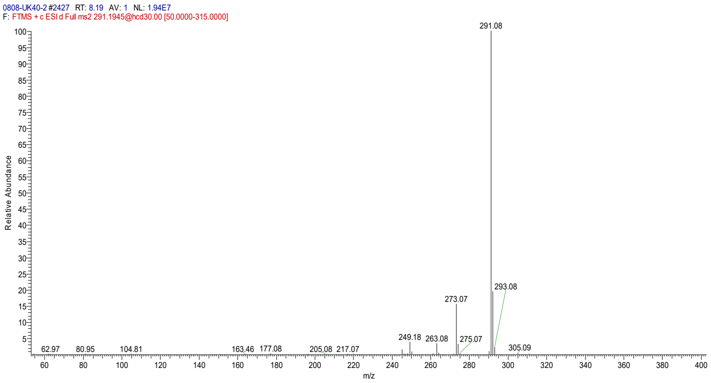


P12

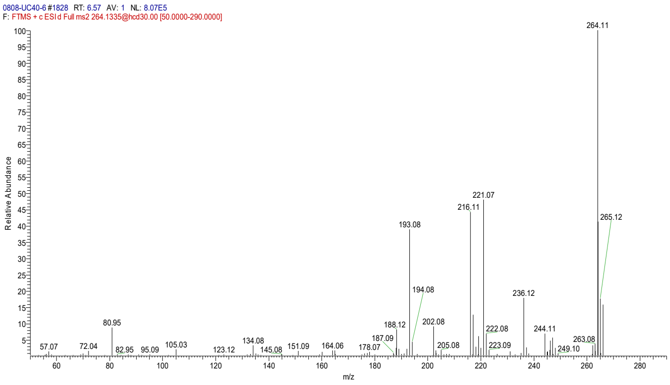


P13

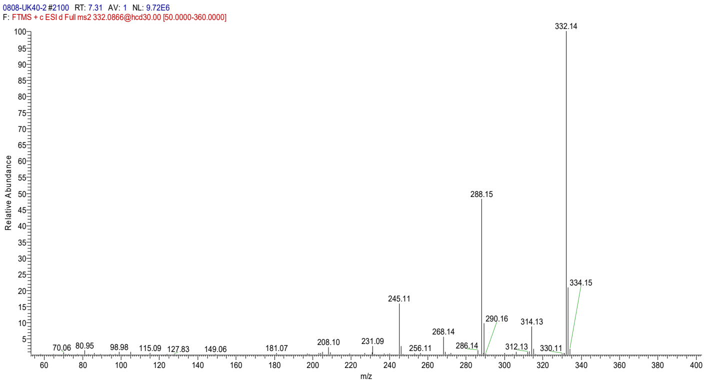


P14

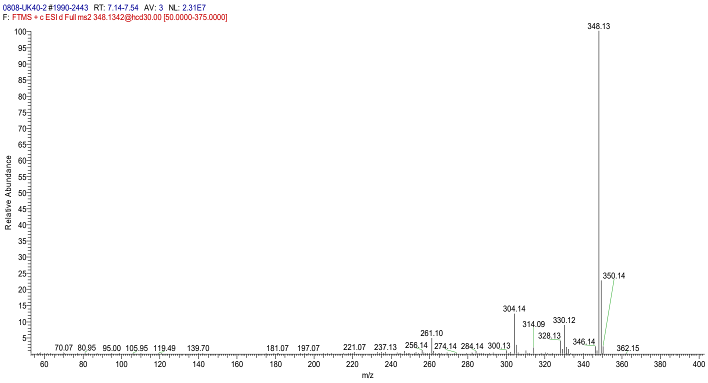


P15

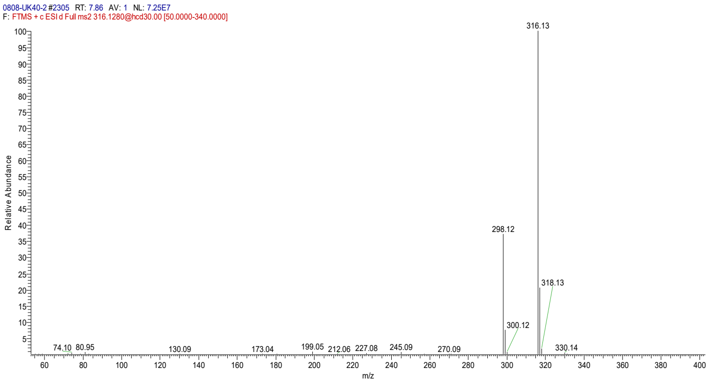


P16

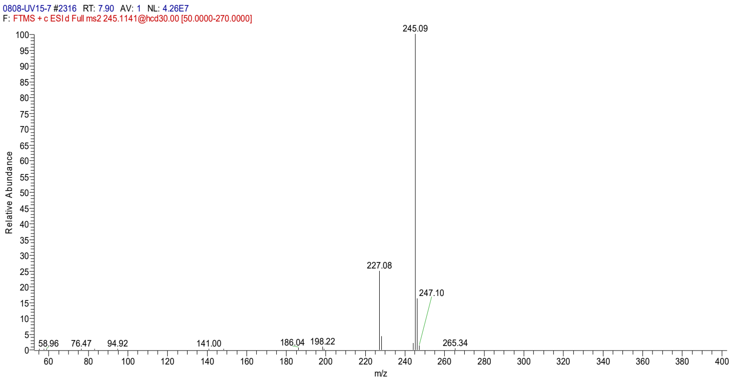


P17

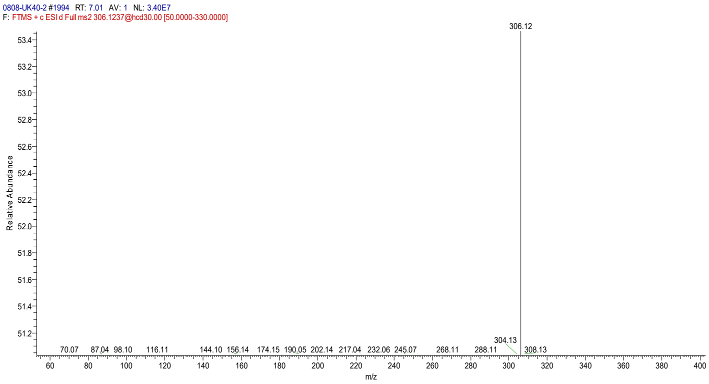


**Figure S3.** Fragment mass spectrometry of CIP-derived DBPs

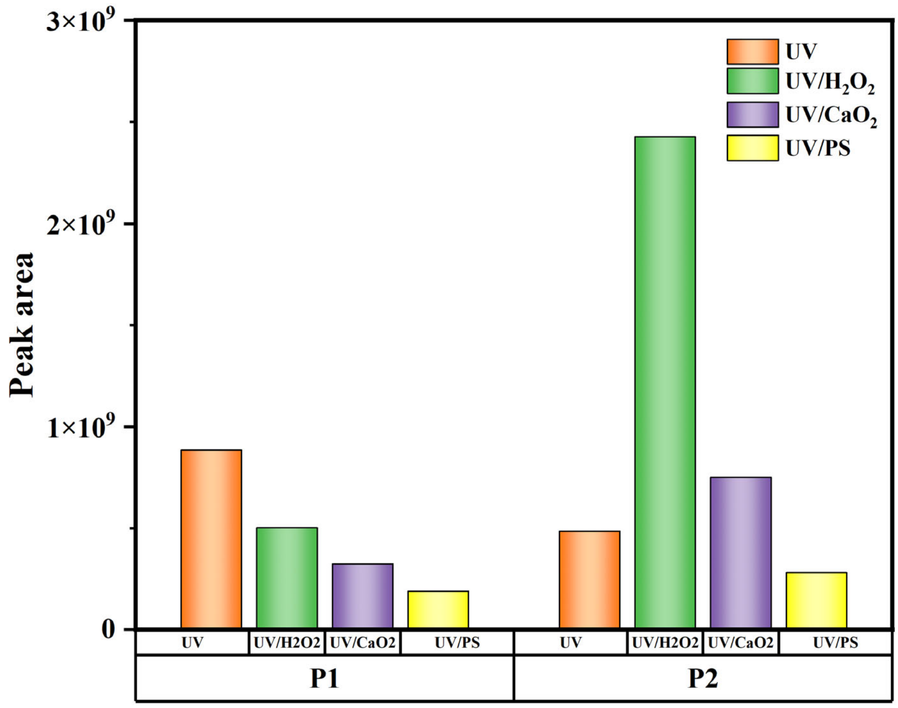


**Figure S4.** Peak area chart of main intermediates P1 and P2 from (a) UV, (b) UV/H2O2, (c) UV/CaO2, (d) UV/PS

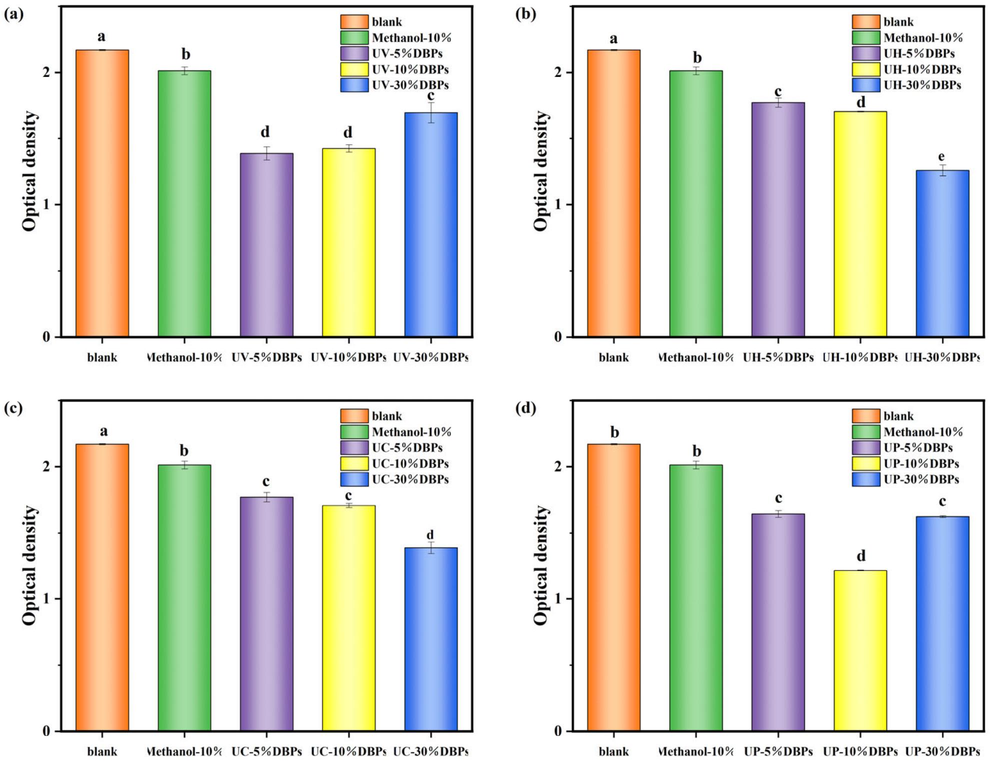

**Figure S5.** Growth inhibition of microalgae on the 12th day of exposure to disinfection by-products, which are derived from (a) UV, (b) UV/$H_2O_2$, (c) UV/$CaO_2$, (d) UV/PS ($p < 0.05$). In figures a–d, different lowercase letters (a, b, c, d) indicate statistically significant differences between groups (P<0.05).

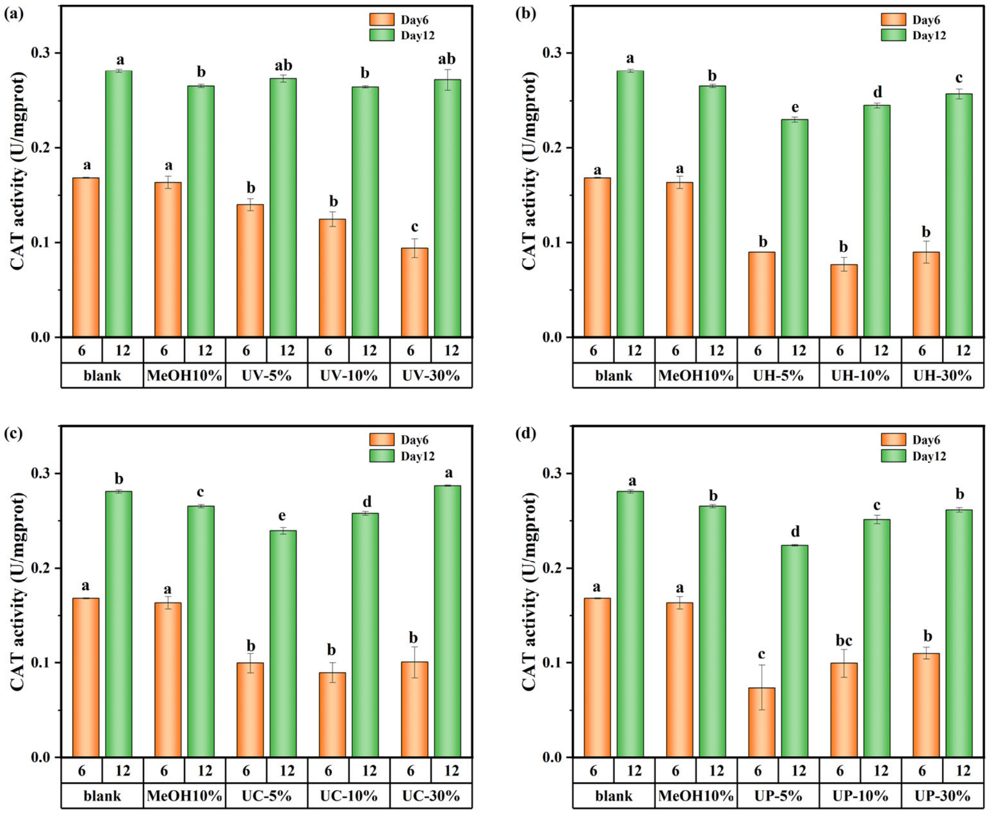


**Figure S6.** CAT activity value of Microcystis aeruginosa exposed to DBPs from (a) UV, (b) UV/$H_2O_2$, (c) UV/$CaO_2$, (d) UV/PS ($p < 0.05$). In figures a–d, different lowercase letters (a, b, c, d) indicate statistically significant differences between groups (P<0.05).
